# Reversible tau hyperphosphorylation in hibernation: a blood biomarker and brain tissue study

**DOI:** 10.1007/s00401-025-02930-2

**Published:** 2025-09-29

**Authors:** Wagner S. Brum, Laia Montoliu-Gaya, Gunnar Brinkmalm, Diana Piotrowska, Elena Camporesi, Carsten Jäger, Helena S. Isaksson, Sven Martin, Jonas Kindberg, Juan Lantero-Rodriguez, João Pedro Ferrari-Souza, Alexis Moscoso, Andrea L. Benedet, Shorena Janelidze, Johan Gobom, Henrik Zetterberg, Oskar Hansson, Eduardo R. Zimmer, Nicholas J. Ashton, Thomas Arendt, Tammaryn Lashley, Jens T. Stieler, Max Holzer, Ole Fröbert, Kaj Blennow

**Affiliations:** 1 Department of Psychiatry and Neurochemistry, Institute of Neuroscience and Physiology, The Sahlgrenska Academy at the University of Gothenburg, Mölndal, Sweden; 2 Graduate Program in Biological Sciences: Biochemistry, Universidade Federal Do Rio Grande Do Sul (UFRGS), Porto Alegre, Brazil; 3 Paul Flechsig Institute, Centre of Neuropathology and Brain Research, University of Leipzig Medical Center, Leipzig, Germany; 4 Department of Neurophysics, Max Planck Institute for Human Cognitive and Brain Sciences, Leipzig, Germany; 5 School of Medical Sciences, Örebro University, Örebro, Sweden; 6 Norwegian Institute for Nature Research, Trondheim, Norway; 7 Department of Wildlife, Fish and Environmental Studies, Swedish University of Agricultural Sciences, Umeå, Sweden; 8 Department of Psychiatry, University of Pittsburgh, Pittsburgh, PA USA; 9 Nuclear Medicine Department and Molecular Imaging Group, Instituto de Investigación Sanitaria de Santiago de Compostela, Travesía da Choupana S/N, Santiago de Compostela, Spain; 10 Clinical Memory Research Unit, Department of Clinical Sciences Malmö, Faculty of Medicine, Lund University, Lund, Sweden; 11 Department of Neurodegenerative Disease, Queen Square Institute of Neurology, University College London, London, UK; 12 UK Dementia Research Institute, University College London, London, UK; 13 Clinical Neurochemistry Laboratory, Sahlgrenska University Hospital, Mölndal, Sweden; 14 Hong Kong Center for Neurodegenerative Diseases, Clear Water Bay, Hong Kong, China; 15 Wisconsin Alzheimer’s Disease Research Center, School of Medicine and Public Health, University of Wisconsin , Madison, WI USA; 16 Memory Clinic, Skåne University Hospital, Malmö, Sweden; 17 Graduate Program in Biological Sciences: Pharmacology and Therapeutics, UFRGS, Porto Alegre, Brazil; 18 Department of Pharmacology, UFRGS, Porto Alegre, Brazil; 19 McGill Centre for Studies in Aging, McGill University, Montreal, QC Canada; 20 Banner Alzheimer’s Institute, Phoenix, AZ USA; 21 Banner Sun Health Research Institute, Sun City, AZ USA; 22 Faculty of Health, Department of Cardiology, Örebro University, Örebro, Sweden; 23 Department of Clinical Medicine, Faculty of Health, Aarhus University, Aarhus, Denmark; 24 Department of Clinical Pharmacology, Aarhus University Hospital, Aarhus, Denmark; 25 Steno Diabetes Center Aarhus, Aarhus University Hospital, Aarhus, Denmark; 26 Paris Brain Institute, ICM, Pitié-Salpêtrière Hospital, Sorbonne University, Paris, France; 27 Neurodegenerative Disorder Research Center, Division of Life Sciences and Medicine, Department of Neurology, Institute On Aging and Brain Disorders, University of Science and Technology of China and First Affiliated Hospital of USTC, Hefei, People’s Republic of China

**Keywords:** Alzheimer’s disease, Tau, Phosphorylated tau, Tauopathies, Hibernation, Plasma biomarkers, Neuropathology

## Abstract

Tau hyperphosphorylation, a key neuropathological feature of tauopathies such as Alzheimer’s disease (AD), also occurs physiologically during mammalian hibernation and is fully reversed upon arousal, offering a unique translational model to study tau metabolism. However, limited data exist on insoluble and soluble tau alterations during hibernation and on patterns of tau fragment concentrations in the hibernating mammalian brain. We quantified tau biomarkers in plasma samples from ten free-ranging brown bears (*Ursus arctos*), captured during both their active summer period and hibernation in the winter, using clinically validated immunoassays and immunoprecipitation mass spectrometry (IP-MS) techniques. We also analyzed brain tissue from ten golden Syrian hamsters (*Mesocricetus auratus*) subjected to induced torpor (hibernation) versus euthermic (non-hibernating) states by quantifying multiple phosphorylated and non-phosphorylated tau peptides with an IP-MS method previously applied in human brain tissue. In brown bears, plasma levels of phosphorylated tau (p-tau) biomarkers p-tau181 and p-tau217 significantly increased during hibernation compared to summer (median increases of 362% and 294% by IP-MS, respectively), with similar increases found with immunoassays. Additional plasma p-tau biomarkers associated with AD pathology, including p-tau205 and p-tau231, were also increased during bear hibernation. In hamster brains, p-tau217, and p-tau231 were similarly elevated during torpor, while tau fragments from the microtubule-binding region (MTBR), associated with tangle aggregation, were not increased. In contrast, brain tissue from *n* = 10 AD patients, analyzed with the same IP-MS method, exhibited striking increases in p-tau (~ 50,000% for p-tau217) and MTBR fragments (~ 20,000% for MTBR tau354-369) compared with *n* = 10 human controls. We show that hibernation-linked tau hyperphosphorylation involves some of the same phospho-sites altered in AD, but occurs without MTBR tau aggregation. This highlights hibernation as a reversible, non-pathological model to study tau biology and mechanisms underlying AD due to its reversibility and lack of tau aggregation despite hyperphosphorylation in key AD tau phospho-sites.

**Supplementary Information:**

The online version contains supplementary material available at 10.1007/s00401-025-02930-2.

## Introduction

The accumulation of microtubule-associated protein tau (MAPT) in intracellular fibrillary deposits is a neuropathological hallmark of tauopathies [1]. In Alzheimer’s disease (AD), the most prevalent tauopathy, the accumulation of misfolded and hyperphosphorylated tau (p-tau) is associated with the onset and progression of cognitive decline [2]. Tau protein undergoes abnormal hyperphosphorylation at multiple sites, forming paired helical filaments that aggregate into neurofibrillary tangles in the cytoplasm, neuropil threads in dendrites, and the neuritic corona of plaques in axons and nerve terminals [1].

Traditionally, tau pathology could only be confirmed through postmortem neuropathological examination. However, recent advances enable in vivo tracking of tau abnormalities. Positron emission tomography (PET) topographically quantifies brain tau tangles, while biofluid-based biomarkers measure soluble tau and phosphorylated tau (p-tau) in cerebrospinal fluid (CSF) and plasma [3].

The development of plasma biomarkers, particularly p-tau, has transformed AD research, offering robust, minimally invasive tools for disease detection and monitoring [4, 5]. Plasma p-tau levels predict Aβ-positivity, correlate with tau deposition assessed by PET or neuropathology, predict cognitive decline, and associate with clinical progression [6, 7]. Specific plasma p-tau variants (i.e., phosphorylated at different epitopes) may increase distinctly in response to different AD pathophysiological processes, but increases in their levels in AD seem to be associated with the presence of Aβ pathology [8–11].

Despite extensive research on tau dysmetabolism in disease, little is known about its alterations under physiological conditions, partly due to the lack of natural models of tau hyperphosphorylation [1, 12]. Mammalian hibernation, an extreme physiological state enabling animals to endure adverse environmental conditions, has been proposed as such a model [1, 12]. During hibernation, synaptic regression occurs alongside reversible PHF-like tau hyperphosphorylation. Upon arousal, hyperphosphorylation reverses as synapses are restored [12]. Despite occurring annually during hibernation seasons, reversible tau hyperphosphorylation does not seem to lead to tau tangle aggregation in the brain, as seen in humans with neurodegenerative disorders, nor to be involved with Aβ plaque deposition [12–14]. Instead, it has been proposed as a neuroprotective mechanism to maintain synaptic integrity [12, 14]. Most studies investigating hibernation-associated tau hyperphosphorylation have employed immunohistochemistry or immunoblotting in brain tissue [12, 14, 15]. However, it is not yet well established whether these changes are reflected by changes in soluble p-tau levels. Tau profiling in brain tissue through the use of direct quantification methods, such as mass spectrometry, has also not yet been explored. Here, we evaluated changes in soluble tau during hibernation and active periods in paired samples from hibernating bears, and performed brain tissue tau profiling in golden Syrian hamsters in a laboratory-induced hibernation protocol versus euthermal controls, and also perform brain tissue analysis of patients with AD vs controls to allow for comparisons between hibernation tau profiles and AD.

## Materials and methods

### Free-ranging brown bears

Blood samples were collected from 10 free-ranging sub-adult brown bears (*Ursus arctos*) in the Dalarna and Gävleborg counties in Sweden with animals age ranging from 2 to 3 years. Bears were equipped with very-high-frequency transmitter implants and global positioning system radiocollars, to enable their tracking during the seasons. The animals were captured between February and March during their hibernation period in the winter and then again between May and June for blood sampling during their active period in the summer, with collection years between 2017 and 2020. Bears were immobilized by darting with an anesthetic while hibernating in their den during the winter with a combination of medetomidine, zolazepam, tiletamine, and ketamine [16]. In the summer, bears were darted from a helicopter during their active period in their natural habitat, using a combination of medetomidine, zolazepam, and tiletamine [16]. While specific collection years vary between each animal, each animal had one blood sample drawn during the summer and the winter from the same year. Blood samples were collected with EDTA tubes, from the jugular vein within 20 min from darting, and centrifuged at 2000 × g for 10 min up to 1 h after sampling, and stored at −70 ºC until further transportation to the Clinical Neurochemistry Laboratory (Sahlgrenska University Hospital, Sweden), where they were stored at −80 ºC. All activities involving animal handling and sampling were carried in compliance with the Swedish laws, under the approval of the Swedish Ethical Committee on Animal Research (C18/15 and C3/16; “Djuretiska nämnden, Uppsala, Sweden”), and studies did not involve endangered or protected species. Field activities were undertaken by personnel of the Scandinavian Brown Bear Research Project (https://www.brownbearproject.com/).

### Bear plasma tau quantification

At the Clinical Neurochemistry Laboratory (Sahlgrenska University Hospital, Sweden), we performed immunoprecipitation mass spectrometry (IP-MS) detection of phosphorylated and non-phosphorylated tau peptides by making a minor adaptation of a previously validated method to the *Ursus arctos* tau sequence [11]. Briefly, to prepare the EDTA plasma samples (1 mL), they were thawed, vortexed for 30 s at 2000 rpm, and centrifuged at 4,000 × g for 10 min. Tau protein extraction was achieved through immunoprecipitation (IP) using Dynabeads M-280 Sheep Anti-Mouse IgG (Thermo Scientific, Cat#11202D), which were cross-linked with antibodies targeting non-phosphorylated tau: HT7 (Thermo Scientific, Cat#MN1000) and BT2 (Thermo Scientific, Cat#MN1010). The IP process was automated using the KingFisher Flex System (Thermo Scientific). Further tau enrichment involved adding 15 µl of 60% perchloric acid (PCA) to the samples. Desalting was carried out with a 96-well SPE plate (Oasis PRiME HLB 96-well µElution Plate, 3 mg sorbent per well, Waters), followed by speed-vac drying of the samples. For tryptic digestion, the dried samples were resuspended in a trypsin solution (Sequencing grade, Promega) at 0.1 µg per sample (2.5 µg/ml in 50 mM AMBIC) and incubated at 37 °C. After 18 h, the reaction was halted by adding trifluoroacetic acid (TFA) to a final concentration of 0.1%, and the samples were lyophilized and stored at −20 °C. Before liquid chromatography–mass spectrometry analysis, the samples were reconstituted in 50 µl of 0.01% TFA and analyzed as singlicates. Mass spectrometry was performed using a hybrid Orbitrap mass spectrometer (Lumos, Thermo Scientific) equipped with an EasySpray nano-ESI ion source. The instrument operated in positive-ion mode with the following parallel reaction monitoring (PRM) scan settings: Activation Type: HCD, Detector Type: Orbitrap, Orbitrap Resolution: 60,000, Scan Range: 250–1200, RF Lens: 30%, Easy-IC: On, Quadrupole Isolation Window: 0.7 m/z. Parameters for Maximum Injection Time, Normalized AGC Target, optimal collision energy, and FAIMS voltage were determined for each peptide experimentally. Endogenous tryptic peptides targeted are listed in Supplementary Tables S1 and S2. Notably, the tau sequence in *Ursus arctos* was conserved except for the region containing the p-tau181 peptide. In humans, this sequence is TPPAPKpTPPSSGEPPK, whereas in *Ursus arctos*, it is TTPSPKpTPPGESGK. A heavy-labeled peptide for the bear-specific sequence was designed, and the mass spectrometry parameters were optimized accordingly. LC–MS data acquisition was conducted using Xcalibur 4.5 and Tune 3.5 software (Thermo Scientific), and data analysis was performed with Skyline software (McCoss Lab, University of Washington). In addition, plasma p-tau181 and p-tau217 were also quantified by research-grade clinically validated immunoassays. Plasma p-tau181 was measured using the Lumipulse plasma p-tau181 assay, developed as a modified version of their FDA-approved Lumipulse G CSF p-Tau181 assay, using the LUMIPULSE G1200, also at the University of Gothenburg [17]. Plasma p-tau217 was quantified using immunoassay (developed by Lilly Research laboratories) on the Meso Scale Discovery platform, at the Clinical Memory Research Unit (Lund University, Sweden), and its clinical validation has been previously described [18]. Before analyses, plasma samples previously stored at -80 ºC freezers were thawed, vortexed and centrifuged at 4000 × g for 10 min. Scientists and technicians were blinded to the subjects’ information, and internal quality controls were used to monitor analyses.

### Golden Syrian hamsters

We included brain tissue data analyzed from *n* = 10 wild-type golden Syrian hamsters (*Mesocricetus auratus*), with animals that were obtained from Janvier Labs at an age of 35 weeks. Torpor was induced in a dedicated climate chamber at the Paul Flechsig Institute, University of Leipzig according to Oklejewicz et al. [19] by exposing the animals to several weeks of short photoperiod as follows: 4 weeks of ‘short day’ (8 h light and 16 h darkness) at room temperature (22 °C) followed by continuous dim red light conditions (LED light with a wavelength > 650 nm) and a reduction of the ambient temperature to 5–6 °C. General locomotor activity was monitored with passive infrared detectors mounted above each cage allowing the discrimination between torpor (periods with > 24 h of inactivity) and euthermic phases [19]. Hamsters that completed at least one torpor cycle and were inactive for at least 24 h were referred as to “torpor”. Hamsters kept under identical conditions but who did not enter torpor were referred as to “eutherm”. Before sacrifice, hibernation state of the animals was confirmed by measurements of body temperature with an IR thermometer, and upon death core, body temperature was determined using thermocouple thermometer with measurements ranging between ∼ 7 °C for torpid animals and ∼ 35 °C for euthermic hamsters. Five animals were sacrificed during euthermy and five in deep hibernation (torpor). All animals were about 11 months old when they were killed by means of an overdose inhalation of isoflurane (5%) combined with an intraperitoneal injection of 200 mg/kg ketamine, preceding perfusion. After euthanasia, a neocortical brain slice was extracted from each animal. The experiments were approved by the Animal Care and Use Committee of the University of Leipzig (TVV 06/19). They conformed to the European Communities Council Directive (86/609/EEC).

### Human brain samples

Post-mortem human brain tissue was obtained from the Queen Square Brain Bank for Neurological Disorders (QSBB), Department of Clinical and Movement Neurosciences, University College London (UCL) Queen Square Institute of Neurology. Frontal cortex samples (Brodmann area 9) from individuals with Alzheimer’s disease (AD; *n* = 10) and age-matched neurologically healthy controls (*n* = 10) were included. All tissue was flash-frozen at − 80 °C on brass plates immediately upon donation to QSBB and subsequently stored at − 80 °C until analysis. The neuropathological assessment of AD cases adhered to the 2012 National Institute on Aging-Alzheimer’s Association (NIA-AA) guidelines, and the clinical diagnosis met National Institute of Neurological and Communicative Disorders and Stroke (NINCDS) criteria for probable AD [20, 21]. Control donors exhibited no documented neurological disease during life. Use of human tissue followed the ethical principles of the Declaration of Helsinki and was approved by regional ethics committees at UCL and the University of Gothenburg.

### Brain tissue tau quantification

Neocortical brain tissue samples (mean ± SD tissue weight 243 ± 22 mg) from the *n* = 10 golden hamsters, *n* = 10 patients with AD, and *n* = 10 control participants were homogenized, and both the soluble [tris(hydroxymethyl)aminomethane (tris)-buffered saline; TBS] and insoluble (sarkosyl insoluble; SI) fractions were extracted, based on a previously described protocol, with further extraction details detailed elsewhere [22, 23]. Resulting supernatants, containing tau soluble forms and small oligomers, were aliquoted and stored at −80 °C. We applied our novel technique combining immunoprecipitation and high-resolution mass spectrometry for the simultaneous detection and quantification of several tau peptides in brain tissue in their phosphorylated and non-phosphorylated form. As our plasma IP-MS tau method, the IP-MS technique for tau quantification in brain tissue has been validated against AD and other tauopathies in humans and was previously described in detail [24]. In brief, 10 µg of total protein for the TBS and 4.5 µg for the SI homogenates were immunoprecipitated using Dynabeads M-280 Sheep Anti-Mouse IgG (Thermo Scientific, Cat#11202D), conjugated with the HT7 (Thermo Scientific, Cat#MN1000) antibody targeting non-phosphorylated tau (targeting tau at amino acids 159–163). Ten microlitres of the human [*U*-^15^N]-protein standards mix (containing 400 fmol protein) were added to each sample. The protein standard mix comprises 0N3R, 1N4R, and 2N4R human tau isoforms. Subsequently, samples were washed and eluted using a magnetic particle processor (KingFisher, Thermo Fisher Scientific). The eluates (100 µl of 0.5% formic acid) were dried in a vacuum centrifuge at room temperature and stored at −80 °C before resuspension in 50 mM AMBIC and overnight (37 °C, light shaking) enzymatic digestion using trypsin (Sequencing grade, Promega) in the amount of 0.1 µg/sample. Additionally, labeled phospho-peptides (100 fmol each) were also added to each sample before trypsinisation. Tryptic peptides were then analyzed using nanoflow (300 nL/min) LC–MS with a Dionex 3000 system coupled to a hybrid quadrupole-orbitrap Q Exactive instrument (both Thermo Fisher Scientific) set to data-dependent acquisition mode, followed by peptide identification and quantification with PEAKS Xpro (Bioinformatic Solutions) and Skyline software, respectively. As the method was developed for human tau, for the hamster analyses, a minor adaptation of was done to match *Mesocricetus auratus* tau sequence, including modification of a customized database to contain both human and hamster protein sequences for peptide identification as well as adding the required peptides to the quantification scheme. Details of the sequences used for tau identification and quantification can be found in Supplementary Tables S3 and S4.

### Statistical analysis

Descriptive statistics are summarized with median and interquartile ranges for continuous variables or counts and percentages for categorical variables. For outlier detection, we set a stringent criterion of 15 median absolute deviations (MAD) above the median. Among plasma quantifications, only one outlier observation with 15.2 MAD was detected, for a plasma p-tau217 summer quantification measured with the MSD immunoassay, with this bear’s results being excluded from analyses only for this assay. In brain tissue analyses, no outliers were observed for human data, and only one outlier was detected for hamsters’ brain tau quantifications, with a high p-tau217 value in the hibernation group in the SI fraction. To evaluate differences in concentrations of bear plasma biomarker levels between the summer and hibernation in the winter, we used the non-parametric Wilcoxon signed-rank test for paired observations. For comparing hamster brain tissue tau levels between euthermy and torpor, we used the Wilcoxon signed-rank test for non-paired observations. To numerically quantify magnitude of differences between hibernation conditions, we computed the percent median change between hibernation (winter for bears, torpor for hamsters) and the reference group (summer for bears, euthermy for hamsters). For visualization, we displayed boxplots with dots corresponding to the individual biomarker values. For bears, for whom paired data were available, individual dots from the same bears linked values from summer and winter. Statistical significance was set at a two-sided alpha of 0.05. No multiple testing correction was carried, and results were interpreted accordingly. All data analyses and visualization was performed using R version 4.2.1 (R Foundation for Statistical Computing; https://www.r-project.org/).

## Results

### Plasma tau biomarkers in hibernating bears

**Fig. 1:**
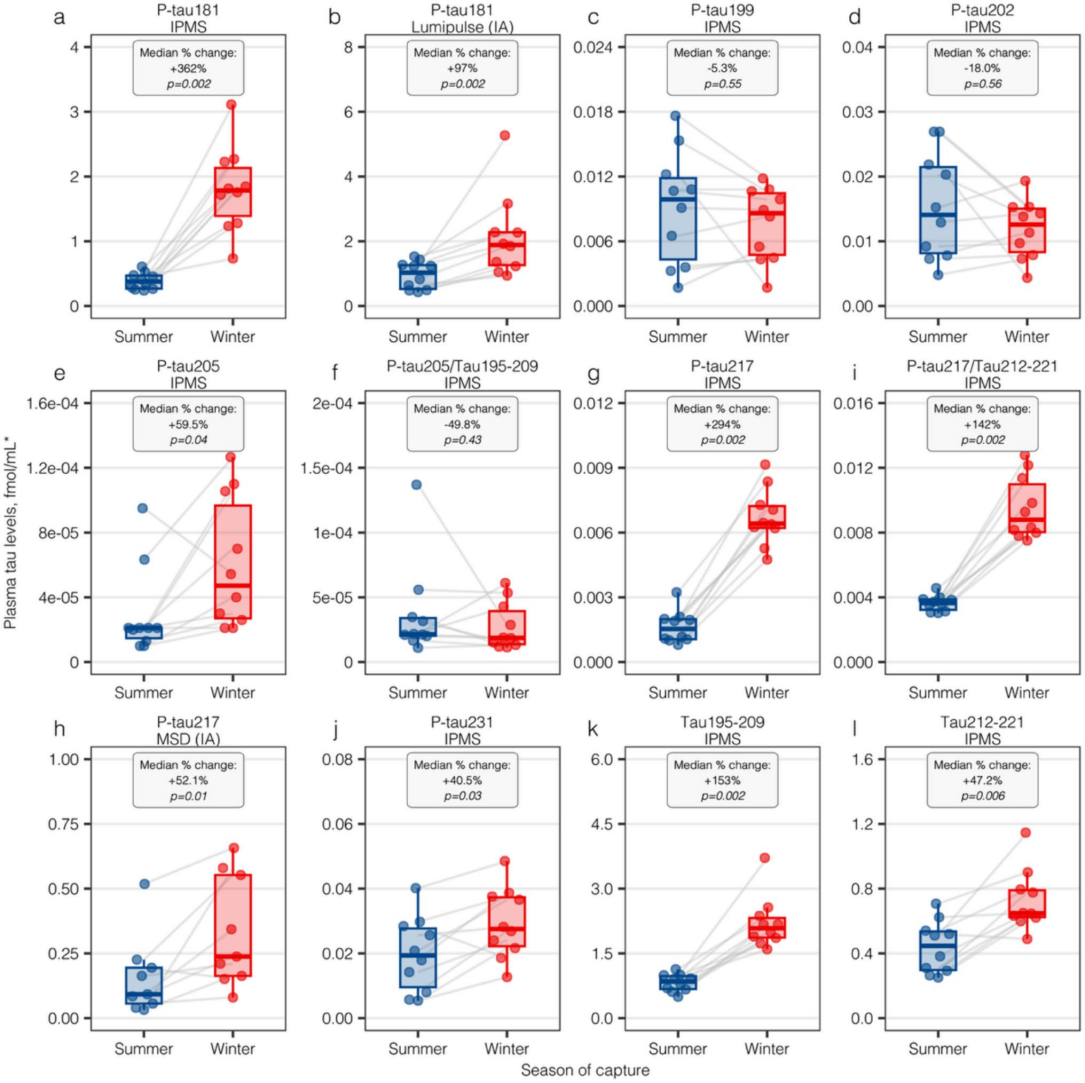
Tau plasma biomarker levels during summer and winter in Swedish brown bears. Dots indicate individual data points for biomarker concentrations on the *y*-axis, and gray lines connect observations between summer (blue) and winter (red), which correspond the *x*-axis groups. Within each specific tau biomarker graph, text boxes indicate the median percent change between summer and winter, as well as the *p *value from a paired-sample non-parametric test. All biomarkers are presented in fmol/mL, with the exception of biomarker ratios (*) which are dimensionless. P-tau: phosphorylated tau; IP-MS: immunoprecipitation mass spectrometry; IA: immunoassay

The bear cohort included 6 females and 4 males, with a median weight of 44.2 kg (IQR, 40.6–49.2 kg) in summer and 40.5 kg (37.5–54.0 kg) in winter. Figure 1 displays tau biomarkers in phosphorylation site order. High increases were observed during hibernation in plasma p-tau181 (IP-MS: median percent-change + 362%, *p* = 0.002; Fig. 1A; IA: + 97%, *p* = 0.002; Fig. 1B). Plasma p-tau199 and p-tau202, however, did not show significant changes (both *p* > 0.05; Fig. 1C-D). A much less pronounced hibernation-linked increase was observed for p-tau205 (+ 59.5%, *p* = 0.044; Fig. 1E), and the p-tau205/tau195-209 ratio was not significantly altered during winter (*p* = 0.43; Fig. 1F). Plasma p-tau217 was also greatly increased during hibernation (IP-MS: + 294%, *p* = 0.002; Fig. 1G; IA: + 52.1%, *p* = 0.01; Fig. 1I), with significant but less pronounced elevations observed for the p-tau217/tau212-221 ratio, also known as %p-tau217 (+ 142%, *p* = 0.002; Fig. 1H). Plasma p-tau231 levels were moderately increased during hibernation (+ 40.5%, *p* = 0.03; Fig. 1J). Interestingly, the non-phosphorylated tau peptides were also substantially increased in the winter compared with summer (tau195-209: + 153%, *p* = 0.002; Fig. 1K; tau212-221: + 47.2%, *p* = 0.006; Fig. 1L).

### Brain tissue tau profiles in hamster hibernation and in Alzheimer’s disease

To complement our findings, we used an IP-MS method developed for brain tissue tau quantification in 10 golden Syrian hamsters (*Mesocricetus auratus*) under an induced hibernation protocol and in AD patients and controls [19, 24]. We quantified the same tau peptides as in plasma (excluding p-tau199, p-tau202, and p-tau/tau ratios) with the addition of two tau microtubule-binding region (MTBR) peptides, and present results in the main text for the SI fraction, which captures the insoluble tau endophenotype.

**Fig. 2:**
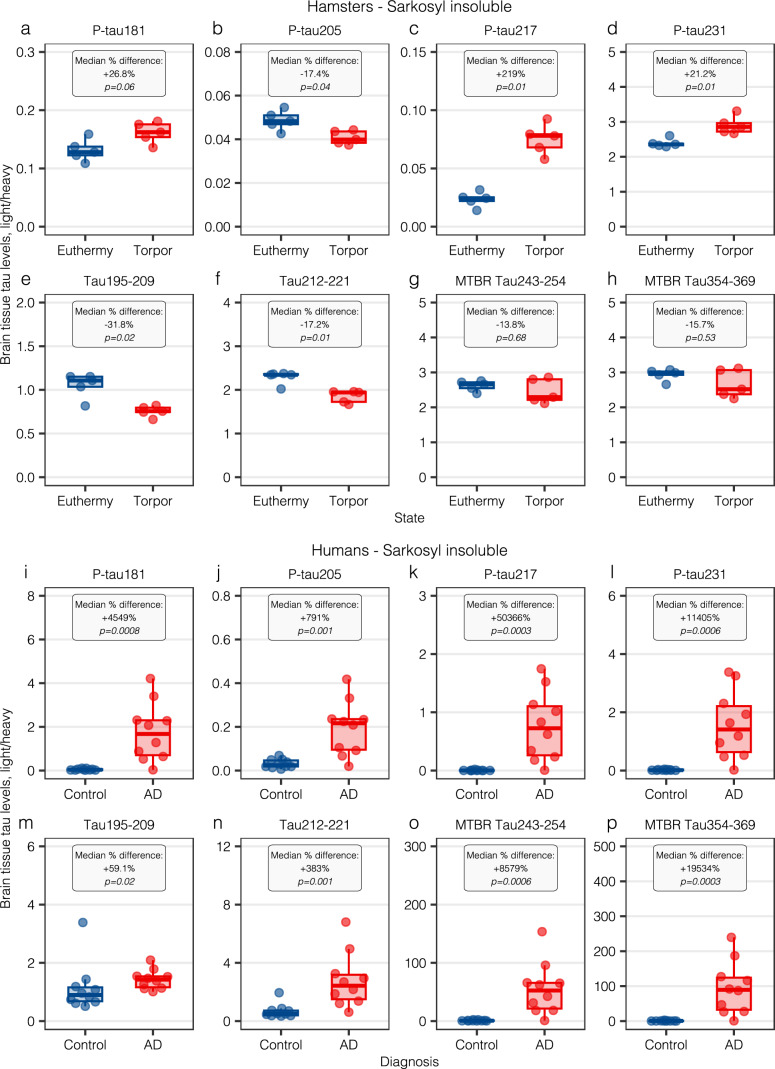
Dots indicate individual data points for tau peptide concentrations in the brain tissue SI fraction for golden Syrian hamsters (panels A–H) and humans with AD and controls (panels I–P). On the x-axis, observations are stratified in euthermy (blue) and torpor/hibernation (red) for hamsters and for controls (blue) and AD (red) groups for humans. Within each specific tau biomarker graph, text boxes indicate the median percent difference between euthermy and torpor or controls and AD, as well as the p-value from a non-parametric test. All biomarkers are presented in the light-to-heavy peptide ratio. SI: sarkosyl insoluble; AD: Alzheimer’s disease; P-tau: phosphorylated tau; IP-MS: immunoprecipitation mass spectrometry; MTBR: microtubule binding region

Hamsters were euthanized during hibernation (torpor, *n* = 5) or euthermy (*n* = 5), and brain tau levels in the SI fraction were compared between groups. Brain p-tau181 levels were slightly higher in the torpor group (median percent difference: + 26.8%, *p* = 0.06, Fig. 2A), with p-tau205 showing a mild decrease (−17.4%, *p* = 0.04, Fig. 2B). P-tau217 exhibited the largest increase (+ 219%, *p* = 0.01, Fig. 2C), with more modest increases in p-tau231 levels (+ 21.2%, *p* = 0.01, Fig. 2D). Tau195-209 (−31.8%, *p* = 0.02, Fig. 2E) and tau212-221 (−17.2%, *p* = 0.01, Fig. 2F) were significantly reduced in the torpor group. Importantly, MTBR tau fragments were not altered between euthermy and hibernation groups (MTBR tau243-254: −13.8%, *p* = 0.68, Fig. 2G; MTBR tau354-369: −15.7%, *p* = 0.53, Fig. 2H).

To compare tau changes in hamster brain tissue during hibernation with pathological changes in AD, we included a sample of *n* = 10 controls (mean age 78.0 years) and *n* = 10 AD patients (mean age 75.8 years). All AD patients (100%) presented advanced levels of neurofibrillary tangle pathology, presenting at autopsy with Braak stage V-VI, with descriptive information displayed in Supplementary Table S5. When analyzing these same peptides in the SI fraction of AD patient brains compared with controls, results were markedly different from hibernation. All p-tau variants analyzed displayed much more dramatic increases in AD compared with controls, with + 4,549% increases for p-tau181 (*p* = 0.0008, Fig. 2I), + 791% for p-tau205 (*p* = 0.001, Fig. 2J), + 50,366% for p-tau217 (*p* = 0.0003, Fig. 2K), and + 11,405% for p-tau231 (*p* = 0.0008, Fig. 2L). Tau195-209 was significantly increased in AD (+ 59.1%, *p* = 0.02, Fig. 2M), and so was tau212-221 (+ 383%, *p* = 0.001, Fig. 2N). MTBR tau fragments, a proxy for neurofibrillary tangle presence, were substantially increased in AD, with + 8,579% increases for MTBR tau243-254 (*p* = 0.0006, Fig. 2O) and + 19,534% increases for MTBR tau354-369 (*p* = 0.0003, Fig. 2P).

In Supp. Fig. S1, results for hamster brain tissue in euthermy and torpor, as well as for controls and AD patients, are displayed for the TBS fraction of brain tissue. For hamsters, TBS results were generally similar to the SI fraction, with more pronounced increases in p-tau217 and also increases in p-tau231 and p-tau181, with no hibernation-linked increases in MTBR peptides and slight reductions in non-phosphorylated tau. For humans, p-tau217 remained substantially increased in the TBS fraction, also showing reductions in non-phosphorylated tau fragments, but MTBR tau fragments, especially MTBR tau354-369, were still increased.

## Discussion

In this study, we used a targeted IP-MS method to quantify phosphorylated and non-phosphorylated tau peptides in plasma from free-ranging brown bears during summer and hibernation and validated the results in hamster brains during torpor and euthermy. Hibernation-linked increases in plasma levels of p-tau181, p-tau205, p-tau217, and p-tau231, with unchanged p-tau199 and p-tau202 levels, resemble a pattern seen in AD patients [11]. We observed the greatest increases in plasma p-tau181 and p-tau217, biomarkers strongly associated with AD neuropathology [18, 25]. These findings align with a previous study on hibernating American black bears that reported, with immunoblotting and immunohistochemistry, neuropathological changes in phospho-sites Thr181, Thr205, Thr217, and Thr231 [14]. While that study reported Ser202 phosphorylation changes and no total-tau alterations, we did not observe p-tau202 changes and did not measure total-tau. Instead, we measured non-phosphorylated fragments (tau195-209 and tau212-221), which were markedly increased during hibernation. While total-tau, measured by immunoassays, is traditionally considered a marker of neurodegeneration, less evidence is available on mass spectrometry-based techniques quantifying smaller non-phosphorylated fragments. Nevertheless, a recent study shows that tau212-221 measured by MS correlates tightly with total-tau measured by the Lumipulse immunoassay (*r* = 0.99) [26], which could explain why we observed increases in tau195-209 and tau212-221 in a similar direction as with their phospho-counterparts, p-tau205 and p-tau217. Given the cleavage peptides undergo before quantification, it is also possible that part of the two non-phosphorylated fragments we measured come from molecules highly phosphorylated at other sites, due to global increases in tau secretion from neurons, a process that may be increased in hibernation. As hibernating animals do not experience neurodegeneration, the observed increases in these non-phosphorylated fragments may reflect hibernation-linked tau processing alterations rather than neurodegeneration [1, 12, 14]. Additionally, the ratios of p-tau205 and p-tau217 to their non-phosphorylated counterparts did not show greater increases than p-tau variants alone. We interpret the changes in plasma p-tau levels during hibernation as indicative of the tau hyperphosphorylation process reported in hibernating animals. While it could be argued that protein levels are increasing due to the hemoconcentration that occurs during hibernation, the previous work from the Scandinavian Brown Bear Project estimates that hemoconcentration is limited to a 10–30% magnitude, which would not account for the > 300% increases we found for plasma p-tau181 and p-tau217 [27]. Additionally, prominent and consistent cerebral Aβ deposition has not been robustly documented in hibernating species—unlike humans where increased p-tau levels are closely associated with Aβ pathology—suggesting that hibernation-linked tau hyperphosphorylation occurs independently of Aβ abnormalities [1].

Our IP-MS findings on brain tau levels in golden Syrian hamsters during hibernation align with bear plasma results obtained via IP-MS and immunoassays. Brain p-tau181, p-tau217, and p-tau231 levels increased, in varying magnitudes, in the torpor group in both TBS and SI fractions (although p-tau181 not significant in SI), consistent with prior brain tissue studies on small hibernators showing high tau phosphorylation rates via immunohistochemistry and immunoblotting [12–14]. Similarly, the observed increases in bear plasma p-tau181 corroborate recent findings in hamsters, where plasma p-tau181 levels were also higher during torpor than arousal when measured with a non-clinically validated biofunctionalization method [28]. This converging evidence highlights the importance of evaluating this hibernation-linked hyperphosphorylation through different species, biological matrices, and analytical methods.

These tau phosphorylation changes may, in part, reflect a passive consequence of lowered body temperature. Planel and colleagues previously proposed that tau hyperphosphorylation under hypothermic conditions results from differential temperature sensitivity of kinases and phosphatases, particularly the inhibition of protein phosphatase 2A [29]. Our findings are compatible with this mechanism, but also align with results from hibernating mammals suggesting an additional hibernation-state-specific regulatory component. In particular, Stieler et al. demonstrated that although low temperatures facilitate tau phosphorylation, tissue from torpid animals shows enhanced phosphate incorporation compared to euthermic controls at similar temperatures, indicating active, regulated mechanisms beyond temperature alone [14]. Thus, hibernation-linked tau phosphorylation likely involves both passive thermodynamic effects and active, reversible processes associated with the hypometabolic state.

It is well established that hibernators do not develop AD-like fibrillar tangle pathology despite seasonal tau hyperphosphorylation [1]. While it has been reported aging bears may form pre-tangle tau aggregates, such formations are rare and not observed in all aged bears [14, 30]. The MTBR domain of tau is a key component of insoluble neurofibrillary tangles, and has even been proposed as a tangle-specific biomarker in biofluids [31, 32]. In brain tissue from AD patients, where tangle pathology is present, MTBR tau fragments showed dramatic increases, compared with controls without AD pathology. In the SI fraction, containing insoluble aggregates, MTBR tau354-369, in particular, was increased by ~ 20,000% and still showed a ~ 238% increase in the soluble TBS fraction, highlighting the substantial tangle burden in AD brains. In hibernating hamsters, however, unlike for p-tau variants, brain levels of MTBR tau243-254 and tau354-369 were not increased compared with euthermy in the SI fraction, and even slightly reduced (-6%) in the TBS fraction. This may be further reflective of the fact that hibernation-linked tau hyperphosphorylation does not lead to tau aggregation with neurofibrillary tangle formation. Additionally, recent evidence suggests that p-tau205, measured in plasma or CSF, may be the p-tau variant with the strongest relation to tau tangles [10, 11, 25]. Interestingly, p-tau205 levels showed no increase in torpor hamster brains, in both SI and TBS fractions, and only modest increases in bear plasma.

Recent work by Lövestam et al. provides compelling evidence that tau hyperphosphorylation at specific residues is sufficient to drive aggregation into Alzheimer-type paired helical filaments. Using 12 phosphomimetic mutations (PAD12) that mimic phosphorylation at sites, including T181, T205, T217, and T231, the authors demonstrated spontaneous in vitro assembly of recombinant full-length tau into filaments structurally indistinguishable from those found in AD brains [33]. These findings establish a mechanistic link between site-specific hyperphosphorylation and the adoption of the AD-specific tau folding, suggesting that tau phosphorylation may be a key driver of filament formation. In our hibernating animal models, we observed reversible phosphorylation at several of these same residues without detectable tau aggregation, suggesting that while phosphorylation can be sufficient under defined experimental conditions and likely also in AD, its capacity to trigger filament formation in vivo may still depend on additional factors not present in hibernation, such as the very long duration of asymptomatic and symptomatic duration of AD, altered clearance, or local biochemical milieu.

Beyond the clear differences in changes in MTBR tau fragments in the brains of AD patients vs controls and lack of changes in hibernating hamster brain tissue, magnitudes of brain tissue phosphorylation were also notably distinct in AD. For instance, in the SI fraction, p-tau217 and p-tau231 were increased by ~ 50,000% in AD vs controls. Even in the TBS fraction, p-tau217 showed an increase of ~ 400% in AD. In contrast, in hibernating hamster brain tissue, p-tau217 showed increases of ~ 200% and ~ 90% in the SI and TBS fractions, respectively, compared with euthermal hamsters. This may underscore a fundamental difference between the regulated, reversible tau phosphorylation observed in hibernation and the pathological tau accumulation and aggregation characteristic of AD. While hamster brain tissue results were remarkably similar between TBS and SI fractions with modest increases in p-tau217 and p-tau231, human results showed much more pronounced increases in all measured tau peptides in SI compared with TBS, possibly suggesting that the shift from soluble phosphorylated tau to insoluble aggregated tau, as observed in AD, does not occur during hibernation. These results, alongside the absence of MTBR tau accumulation in torpor, even in the SI fraction, supports the notion that hibernation-associated hyperphosphorylation does not progress to tangle formation, further suggesting hibernation as a non-pathological model for studying tau biology.

Interestingly, the non-phosphorylated tau195-209 and tau212-221 peptides were decreased by around ~ 20–30% in the hamster brain tissue torpor group. This reduction in non-phospho-peptides was also observed for TBS results in AD brains compared with controls. Unlike with plasma levels, which reflect soluble tau processing in a more dynamic way, the direct quantification of tau in the brain tissue may allow for interpreting that these decreases could be at least partly attributed to increases in phosphorylation rates. The reduction in these non-phosphorylated peptides in brain tissue, combined with their increase in blood levels and the slight decreases in non-phosphorylated MTBR tau fragments, may reflect uncharacterized aspects of tau processing during hibernation.

This work is not exempt from limitations, mostly associated with the high complexity of carrying out longitudinal monitoring in free-ranging animals. Our sample size for hibernators (bears: *n* = 10; hamsters: *n* = 10) is relatively small, but similar to that of other studies in the interface between AD pathology and hibernation [12, 14, 30]. Also, due to ethical considerations and animal welfare, sampling was restricted to plasma and limited to two occasions, preventing us from quantifying tau proteins in the CSF and from conducting a more detailed repeated-measures analysis of p-tau dynamics throughout the year. While older bears may serve as a more suitable model for aging human disease, the sub-adult (2–3 years old) bears in our study still exhibited marked changes in plasma p-tau levels. Also, studying older free-ranging bears presents significant challenges, including increased potential for comorbidities and, especially, greater risks to the research team. Brain tissue is not collected within the Scandinavian Brown Bear Research Project, preventing neuropathological analyses in bears, and hamster plasma analysis was not possible due to the high-volume needed by the IP-MS plasma method, which is considerably larger than the small volume of blood usually collected from each hamster. When using methods based on centrifugation and relative detergent solubility, it is possible that some degree of cross-contamination may occur across fractions. Thus, we cannot exclude that the low levels measure here in hamster brain may be tau proteins that pelleted or fragments of non-aggregated insoluble tau (previously reported in hibernation), despite not being assembled in AD-like NFTs [14]. At the same time, it is evident from the results of our study that the differences for the key p-tau species measured (the best example being pTau217) change in the same direction as in AD and hamster brain in both SI and TBS fractions (as well as AD CSF/plasma from the literature), and even in plasma from hibernating bears. However, other tau variants which would be expected in the presence of NFT pathology, such as the MTBR species and p-tau205, do not change. We understand that this speaks against the fact that cross-contamination could be driving the results, and that there is a consistent between-species consistent biological phenomenon of tau phosphorylation during hibernation.

To the best of our knowledge, our study provides the first evidence that hibernation-linked tau hyperphosphorylation is reflected in increases in plasma p-tau as measured by validated assays, at the same phospho-sites which are increased in AD patients, as well as the first mass-spectrometry characterization of tau processing in brain tissue during hibernation. The pattern of brain and plasma tau levels is consistent with the lack of tangle formation during hibernation. Further translational studies of this phenomenon may provide insights into the biology of p-tau as well as identify novel strategies to prevent p-tau accumulation in the human brain, taking into account the dynamics of both soluble and insoluble tau. Our results also emphasize the need for more research on hibernation as a translational model for advancing our knowledge on aging-related human diseases.

## Supplementary Information

Below is the link to the electronic supplementary material.

Supplementary file1 (DOCX 459 KB)

## Data Availability

The datasets used and/or analyzed during this study are available from the corresponding author upon reasonable request.

## References

[1] Arendt T, Stieler JT, Holzer M (2016) Tau and tauopathies. Brain Res Bull 126:238–292 27615390 10.1016/j.brainresbull.2016.08.018

[2] Ballatore C, Lee VMY, Trojanowski JQ (2007) Tau-mediated neurodegeneration in Alzheimer’s disease and related disorders. Nat Rev Neurosci 8(9):663–672 17684513 10.1038/nrn2194

[3] Hansson O (2021) Biomarkers for neurodegenerative diseases. Nat Med 27(6):954–963 34083813 10.1038/s41591-021-01382-x

[4] Karikari TK, Ashton NJ, Brinkmalm G, Brum WS, Benedet AL, Montoliu-Gaya L et al (2022) Blood phospho-tau in Alzheimer disease: analysis, interpretation, and clinical utility. Nat Rev Neurol 18(7):400–418 35585226 10.1038/s41582-022-00665-2

[5] Therriault J, Brum WS, Trudel L, Macedo AC, Bitencourt FV, Martins-Pfeifer CC, Nakouzi M, Pola I, Wong M, Kac PR, Real AP, Witherow C, Karikari TK, Moscoso A, Zimmer ER, Schöll M, Pascoal T, Benedet AL, Ashton NJ, Schindler SE, Zetterberg H, Blennow K, Rosa-Neto P (2025) Blood phosphorylated tau for the diagnosis of Alzheimer's disease: a systematic review and meta-analysis. Lancet Neurol 24(9), 740–752. 10.1016/S1474-4422(25)00227-3 40818474 10.1016/S1474-4422(25)00227-3

[6] Palmqvist S, Tideman P, Cullen N, Zetterberg H, Blennow K, Alzheimer’s Disease Neuroimaging Initiative et al (2021) Prediction of future Alzheimer’s disease dementia using plasma phospho-tau combined with other accessible measures. Nat Med 27(6):1034–1042 34031605 10.1038/s41591-021-01348-z

[7] Therriault J, Ashton NJ, Pola I, Triana-Baltzer G, Brum WS, Di Molfetta G et al (2024) Comparison of two plasma p-tau217 assays to detect and monitor Alzheimer’s pathology. EBioMedicine 102:105046 38471397 10.1016/j.ebiom.2024.105046 PMC10943661

[8] Ashton NJ, Brum WS, Di Molfetta G, Benedet AL, Arslan B, Jonaitis E et al (2024) Diagnostic accuracy of a plasma phosphorylated tau 217 immunoassay for Alzheimer disease pathology. JAMA Neurol. 10.1001/jamaneurol.2023.5319 38252443 10.1001/jamaneurol.2023.5319 PMC10804282

[9] Ashton NJ, Janelidze S, Mattsson-Carlgren N, Binette AP, Strandberg O, Brum WS et al (2022) Differential roles of Aβ42/40, p-tau231 and p-tau217 for Alzheimer’s trial selection and disease monitoring. Nat Med. 10.1038/s41591-022-02074-w 36456833 10.1038/s41591-022-02074-w PMC9800279

[10] Lantero-Rodriguez J, Montoliu-Gaya L, Benedet AL, Vrillon A, Dumurgier J, Cognat E et al (2024) CSF p-tau205: a biomarker of tau pathology in Alzheimer’s disease. Acta Neuropathol 147(1):12 38184490 10.1007/s00401-023-02659-w PMC10771353

[11] Montoliu-Gaya L, Benedet AL, Tissot C, Vrillon A, Ashton NJ, Brum WS et al (2023) Mass spectrometric simultaneous quantification of tau species in plasma shows differential associations with amyloid and tau pathologies. Nat Aging 27:1–9 10.1038/s43587-023-00405-1 PMC10275761 37198279

[12] Arendt T, Stieler J, Strijkstra AM, Hut RA, Rüdiger J, Van der Zee EA et al (2003) Reversible paired helical filament-like phosphorylation of tau is an adaptive process associated with neuronal plasticity in hibernating animals. J Neurosci 23(18):6972–6981 12904458 10.1523/JNEUROSCI.23-18-06972.2003 PMC6740664

[13] Bullmann T, Feneberg E, Kretzschmann TP, Ogunlade V, Holzer M, Arendt T (2019) Hibernation impairs odor discrimination - implications for Alzheimer’s disease. Front Neuroanat 13:69 31379517 10.3389/fnana.2019.00069 PMC6646461

[14] Stieler JT, Bullmann T, Kohl F, Tøien Ø, Brückner MK, Härtig W et al (2011) The physiological link between metabolic rate depression and Tau phosphorylation in Mammalian hibernation. PLoS ONE 6(1):e14530 21267079 10.1371/journal.pone.0014530 PMC3022585

[15] Härtig W, Klein C, Brauer K, Schüppel KF, Arendt T, Brückner G et al (2000) Abnormally phosphorylated protein tau in the cortex of aged individuals of various mammalian orders. Acta Neuropathol 100(3):305–312 10965801 10.1007/s004010000183

[16] Evans AL, Sahlén V, Støen OG, Fahlman Å, Brunberg S, Madslien K et al (2012) Capture, anesthesia, and disturbance of free-ranging brown bears (Ursus arctos) during Hibernation. PLoS ONE 7(7):e40520 22815757 10.1371/journal.pone.0040520 PMC3398017

[17] Wilson EN, Young CB, Ramos Benitez J, Swarovski MS, Feinstein I, Vandijck M et al (2022) Performance of a fully-automated Lumipulse plasma phospho-tau181 assay for Alzheimer’s disease. Alzheimers Res Ther 14(1):172 36371232 10.1186/s13195-022-01116-2 PMC9652927

[18] Palmqvist S, Janelidze S, Quiroz YT, Zetterberg H, Lopera F, Stomrud E et al (2020) Discriminative accuracy of plasma phospho-tau217 for Alzheimer disease vs other neurodegenerative disorders. JAMA 324(8):772–781 32722745 10.1001/jama.2020.12134 PMC7388060

[19] Oklejewicz M, Daan S, Strijkstra AM (2001) Temporal organisation of hibernation in wild-type and tau mutant Syrian hamsters. J Comp Physiol B 171(5):431–439 11497131 10.1007/s003600100193

[20] McKhann G, Drachman D, Folstein M, Katzman R, Price D, Stadlan EM (1984) Clinical diagnosis of Alzheimer’s disease. Neurology 34(7):939–939 6610841 10.1212/wnl.34.7.939

[21] Montine TJ, Phelps CH, Beach TG, Bigio EH, Cairns NJ, Dickson DW et al (2012) National Institute on Aging–Alzheimer’s Association guidelines for the neuropathologic assessment of Alzheimer’s disease: a practical approach. Acta Neuropathol 123(1):1–11 22101365 10.1007/s00401-011-0910-3 PMC3268003

[22] Camporesi E, Lashley T, Gobom J, Lantero-Rodriguez J, Hansson O, Zetterberg H et al (2021) Neuroligin-1 in brain and CSF of neurodegenerative disorders: investigation for synaptic biomarkers. Acta Neuropathol Commun 9(1):19 33522967 10.1186/s40478-021-01119-4 PMC7852195

[23] Sahara N, Kimura T (2018) Biochemical properties of pathology-related tau species in tauopathy brains: an extraction protocol for tau oligomers and aggregates. Methods Mol Biol 1779:435–445 29886548 10.1007/978-1-4939-7816-8_26

[24] Lantero-Rodriguez J, Camporesi E, Montoliu-Gaya L, Gobom J, Piotrowska D, Olsson M et al (2024) Tau protein profiling in tauopathies: a human brain study. Mol Neurodegener 19(1):54 39026372 10.1186/s13024-024-00741-9 PMC11264707

[25] Montoliu-Gaya L, Alosco ML, Yhang E, Tripodis Y, Sconzo D, Ally M et al (2023) Optimal blood tau species for the detection of Alzheimer’s disease neuropathology: an immunoprecipitation mass spectrometry and autopsy study. Acta Neuropathol 147(1):5 38159140 10.1007/s00401-023-02660-3 PMC10757700

[26] Blennow K (2025) Neurodegeneration and acute neuronal injury drastically skew the p-tau217/non-p217 ratio in both CSF and plasma regardless of analytical technique – a mass spectrometry and Simoa study. Alzheimers Dement 20(Suppl 8):e094606

[27] Welinder KG, Hansen R, Overgaard MT, Brohus M, Sønderkær M, von Bergen M et al (2016) Biochemical foundations of health and energy conservation in hibernating free-ranging Subadult Brown Bear Ursus arctos. J Biol Chem 291(43):22509–22523 27609515 10.1074/jbc.M116.742916 PMC5077189

[28] León-Espinosa G, Murillo AMM, Turegano-Lopez M, DeFelipe J, Holgado M (2024) Phosphorylated Tau at T181 accumulates in the serum of hibernating Syrian hamsters and rapidly disappears after arousal. Sci Rep 14(1):20562 39232030 10.1038/s41598-024-71481-5 PMC11375040

[29] Planel E, Miyasaka T, Launey T, Chui DH, Tanemura K, Sato S et al (2004) Alterations in glucose metabolism induce hypothermia leading to Tau hyperphosphorylation through differential inhibition of kinase and phosphatase activities: implications for Alzheimer’s disease. J Neurosci 24(10):2401–2411 15014115 10.1523/JNEUROSCI.5561-03.2004 PMC6729502

[30] Cork LC, Powers RE, Selkoe DJ, Davies P, Geyer JJ, Price DL (1988) Neurofibrillary tangles and senile plaques in aged bears. J Neuropathol Exp Neurol 47(6):629–641 3171607 10.1097/00005072-198811000-00006

[31] Fitzpatrick AWP, Falcon B, He S, Murzin AG, Murshudov G, Garringer HJ et al (2017) Cryo-EM structures of tau filaments from Alzheimer’s disease. Nature 547(7662):185–190 28678775 10.1038/nature23002 PMC5552202

[32] Horie K, Salvadó G, Barthélemy NR, Janelidze S, Li Y, He Y et al (2023) CSF MTBR-tau243 is a specific biomarker of tau tangle pathology in Alzheimer’s disease. Nat Med 29(8):1954–1963 37443334 10.1038/s41591-023-02443-z PMC10427417

[33] Lövestam S, Wagstaff JL, Katsinelos T, Freund SM, Goedert M, Scheres SH. Twelve phosphomimetic mutations induce the assembly of recombinant full-length human tau into paired helical filaments. eLife [Internet]. 2025 Jan 16 [cited 2025 July 9];14. Available from: https://elifesciences.org/reviewed-preprints/104778 10.7554/eLife.104778 PMC13189620 42159330

