# Supplementary material for: Reversible tau hyperphosphorylation in hibernation: a blood biomarker and brain tissue study
Source: Acta Neuropathol. 2025 Sep 29;150(1):36. doi: 10.1007/s00401-025-02930-2 (PMC12479634; doi:10.1007/s00401-025-02930-2)
Supplement: Supplementary file 1 — Supplementary file1 (DOCX 459 KB) [file 401_2025_2930_MOESM1_ESM.docx]

## Supplementary Table 1. Tryptic tau plasma *Ursus Arctos* peptides targeted in the study.

| **Phospho site** | **Peptide aa positions** | **Target peptide sequence** | **Charge state** | ***m/z*** | **NCE [%]** | **FAIMS CV (V)** |
| --- | --- | --- | --- | --- | --- | --- |
| T181 | 175-190 | TTPSPK[pT]PPGESGK | 3 | 556.6062 | 25 | -70 |
| - | 195-209 | SGYSSPGSPGTPGSR | 2 | 697.3208 | 25 | -50 |
| S199 | 195-209 | SGYS[pS]PGSPGTPGSR | 2 | 737.3039 | 25 | -50 |
| S202 | 195-209 | SGYSSPG[pS]PGTPGSR | 2 | 737.3039 | 25 | -50 |
| T205 | 195-209 | SGYSSPGSPG[pT]PGSR | 2 | 737.3039 | 25 | -50 |
| - | 212-221 | TPSLPTPPTR | 2 | 533.7982 | 25 | -70 |
| T217 | 212-221 | TPSLP[pT]PPTR | 2 | 573.7814 | 27 | -60 |
| T231 | 225-240 | KVAVVR[pT]PPKSPSSAK | 3 | 577.9887 | 30 | -60 |

Respective dominant charge state, monoisotopic m/z value, optimal normalized collision energy (NCE) for higher energy collision-induced dissociation (HCD) and optimal compensation voltage (CV) used for FAIMS for each peptide are shown.

## Supplementary Table 2. Heavy standards used for the nomalization of quantified *Ursus Arctos* plasma peptides.

| **Phospho site** | **Peptide aa positions** | **Target peptide sequence** | ***m/z*** | **Spiked in per sample (fmol)** |
| --- | --- | --- | --- | --- |
| T181 | 175-190 | TPPAPK[pT]PPSSGEPP**K** | 558.6129 | 1 |
| - | 195-209 | SGYSSPGSPGTPGS**R** | 702.325 | 0.5 |
| S199 | 195-209 | SG**Y**S[pS]PGSPGTPGSR | 742.3175 | 1 |
| S202 | 195-209 | SGYSSPG[pS]PGTPGS**R** | 742.3081 | 0.5 |
| T205 | 195-209 | SG**Y**SSPGSPG[pT]PGSR | 742.3175 | 0.1 |
| - | 212-221 | TPSLPTPPT**R** | 538.802 | 1 |
| T217 | 212-221 | TPSLP[pT]PPT**R** | 578.786 | 0.5 |
| T231 | 225-240 | **K**VAVVR[pT]PPKSPSSA**K** | 582.0022 | 1 |

Respective monoisotopic m/z value, heavy labelling and spiked in concentrations are shown for each peptide. The heavy labeled aminoacids are marked in bold. Dominant charge state, optimal normalized collision energy (NCE) for higher energy collision-induced dissociation (HCD) and optimal compensation voltage used for FAIMS are the same as for the corresponding light peptides shown in Supplementary Table 1.

## Supplementary Table 3. Tryptic tau *Mesocricetus auratus* peptides targeted in the brain study.

| **Phospho site** | **Peptide aa positions** | **Peptide sequence** | **Charge state** | ***m/z*** | **NCE [%]** |
| --- | --- | --- | --- | --- | --- |
| T181* | 166-180* | TTPSPK[pT]PPGSGETPK | 3 | 554.5976 | 25 |
| - | 195-209 | SGYSSPGSPGTPGSR | 2 | 697.3208 | 20 |
| T205 | 195-209 | SGYSSPGSPG[pT]PGSR | 2 | 737.3039 | 25 |
| - | 212-221 | TPSLPTPPTR | 2 | 533.7982 | 20 |
| T217 | 212-224 | TPSLP[pT]PPTREPK | 3 | 500.9201 | 25 |
| T231 | 225-240 | KVAVVR[pT]PPKSPSSAK | 3 | 577.9887 | 25 |
| - | 243-254 | LQTAPVPMPDLK | 2 | 655.3629 | 22 |
| - | 354-369 | IGSLDNITHVPGGGNK | 3 | 526.9460 | 25 |

Respective dominant charge state, monoisotopic m/z value, and employed normalized collision energy (NCE) for higher energy collision-induced dissociation (HCD) for each peptide are shown.

* Apart from having some amino acids substituted, the amino acid positions for this peptide in *Mesocricetus auratus* differs from that of the human sequence, where the corresponding stretch is 175-190. For the same reason the position of the phosphorylation in *Mesocricetus auratus* is amino acid 172. For simplicity in the main text, we decided to keep the human numbering for the phosphorylation site.

## Supplementary Table 4. Heavy standards used for the nomalization of quantified peptides in the *Mesocricetus auratus* brain study.

| **Phospho site** | **Peptide aa positions** | **Target peptide sequence** | ***m/z*** | **Spiked in per sample (fmol)** |
| --- | --- | --- | --- | --- |
| T181* | 175-190* | TPPAP**K**[pT]PPSSGEPP**K** | 561.9490 | 100 |
| - | 195-209 | SGYSSPGSPGTPGSR-[*U-*^15^N] | 706.2941 | 400 |
| T205 | 195-209 | SGYSSPGSPG[pT]PGS**R** | 742.3081 | 100 |
| - | 212-221 | TPSLPTPPTR | 540.2789 | 400 |
| T217 | 212-224 | TPSLP[pT]PPT**R**EP**K** | 506.9276 | 100 |
| T231 | 225-240 | KVAVV**R**[pT]PP**K**SPSSA**K** | 586.6676 | 100 |
| - | 243-254 | LQTAPVPMPDLK-[*U-*^15^N] | 578.786 | 400 |
| - | 354-369 | IGSLDNITHVPGGGNK-[*U-*^15^N] | 582.0022 | 400 |

Respective monoisotopic m/z value, heavy labelling and spiked in concentrations are shown for each peptide. The heavy labeled amino acids are marked in bold, except when the whole peptide was uniformly labelled with ^15^N [*U-*^15^N]. Charge state, and collision energy settings were the same as for the corresponding light peptides shown in Supplementary Table 3.

* The brain derived peptide 166-180-pT172 from *Mesocricetus auratus* was normalized to the heavy labeled peptide with corrsponding human sequence 175-190-pT181.

## Supplementary Table 5. Demographic information for the clinical post-mortem cohort.

|  | Controls  (n=10) | AD  (n=10) |
| --- | --- | --- |
| Female, n (%) | 2 (20%) | 6 (60%) |
| Age at death, years | 78.0 (7.0) | 75.8 (6.7) |
| Post-mortem delay, hours | 56.0 (30.6) | 58.6 (23.1) |
| Braak stages 0/I-II/III-IV/V-VI | 2/4/4/0 | 0/0/0/10 |
| Thal phases 0/1–2/3/4–5 | 2/3/4/1 | 0/0/0/10 |

Data are presented as n (%) for categorical variables and as mean (SD) for continuous variables. For Braak stages and Thal phases, the number of individuals in each category is presented. AD = Alzheimer’s disease.

**Supplementary Figure 1. Tau biomarker levels in the TBS fraction of golden Syrian hamsters and human brain tissue.**


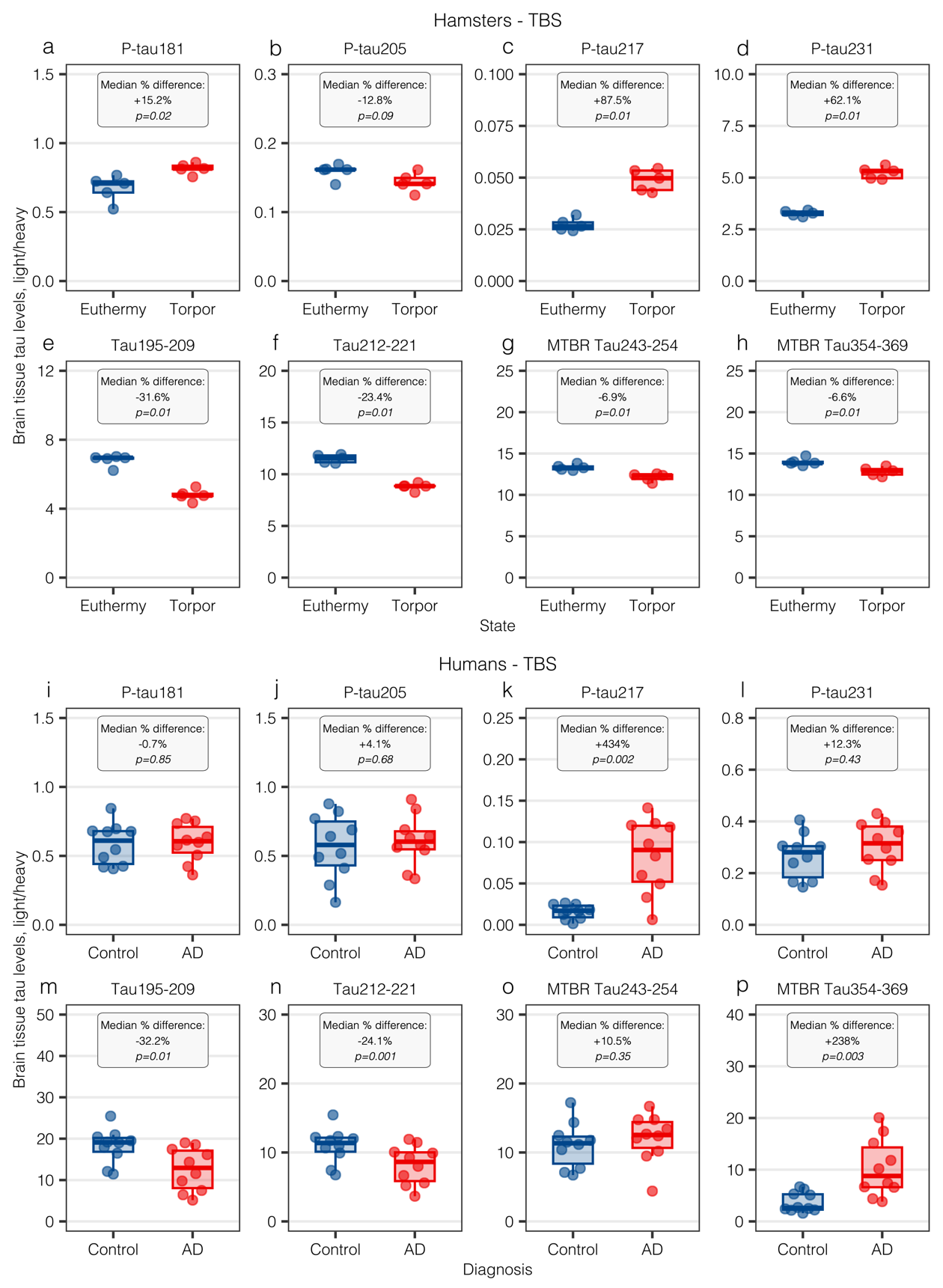


Dots indicate individual data points for tau peptide concentrations in the tris-buffered saline (TBS) fraction of brain tissue of hamsters and humans (Y-axis). On the x-axis, observations are stratified in euthermy (blue) and torpor/hibernation (red) for panels A-H and in controls (blue) and Alzheimer’s disease (AD; red) for panels I-P. Within each specific tau biomarker graph, text boxes indicate the median percent difference between euthermy and torpor or between controls and AD, as well as the p-value from a non-parametric test. All biomarkers are presented in the light-to-heavy peptide ratio. P-tau: phosphorylated tau; IP-MS: immunoprecipitation mass spectrometry; MTBR: microtubule binding region; TBS: tris-buffered saline.
